# The Experiences and Perspective of Partners of Men With Prostate Cancer in Ireland: A Qualitative Descriptive Study

**DOI:** 10.1002/nop2.70585

**Published:** 2026-05-05

**Authors:** Seidu Mumuni, Claire O'Donnell, Owen Doody

**Affiliations:** ^1^ School of Nursing and Midwifery University of Limerick Limerick Ireland; ^2^ School of Nursing and Midwifery, Health Research Institute University of Limerick Limerick Ireland

**Keywords:** caregiving, couple‐centred care, LGBTQ+, partners' experiences, prostate cancer, psychosocial support, qualitative research

## Abstract

**Background:**

Prostate cancer significantly impacts not only the individuals diagnosed but also their partners, who frequently undertake extensive caregiving responsibilities. Despite their crucial role, the experiences and needs of partners remain underrepresented in cancer care literature and policy, particularly in Ireland.

**Methods:**

A qualitative descriptive study exploring the lived experiences of nine partners of men with prostate cancer in Ireland was employed. Semi‐structured interviews were conducted in person, by phone, or virtually. Data were analysed using Braun and Clarke's thematic analysis, supported by NVivo software, and interpreted through Meleis' Transition Theory.

**Results:**

Five major themes emerged: (1) Invisible Needs: Experience in Care, (2) Alone Together: Missing Couple‐Centred Support, (3) Knowledge Deficit and Under preparedness, (4) Hesitancy, Silence and Emotional Restraints and (5) The Cost of Caring: Strain, Self‐Care and Advocacy. Participants highlighted critical issues of marginalisation, emotional burden, under preparedness, and lack of inclusion for a specific group within a normal conventional care setting, which are precisely the experiences that need to be understood and addressed, not dismissed as representing a minority.

**Conclusion:**

Partners of men with prostate cancer, especially those from LGBTQ+ communities, face significant psychosocial challenges that are often overlooked by the healthcare system. Findings underscore the urgent need for inclusive, couple‐centred support models and tailored educational interventions. Enhancing caregiver involvement and wellbeing through systemic and policy‐level changes could improve outcomes for both patients and their partners.

**Patient and Public Involvement Statement:**

Patients and members of the public were not involved in the design or conduct of this research. Although participants contributed their perspectives as part of the qualitative study, there was no formal PPI in shaping the research process. The authors recognise the importance of involving patients and the public in co‐designing research and will consider this in future studies.

## 1 Introduction

Prostate cancer remains one of the most diagnosed cancers among men globally, imposing not only a significant physical burden on patients but also a substantial emotional and psychological stress on their intimate partners (Gandaglia et al. 2021; Barsouk et al. 2020). As the disease progresses through its various stages (thus from diagnosis to treatment and survivorship), partners often assume demanding caregiving roles and responsibilities that extend beyond emotional support to include medical management, advocacy and household responsibilities (Irish Cancer Society [ICS] 2020; Wang et al. 2022). Studies show that partners of prostate cancer patients experience a range of sexual, relational and logistical challenges, which tend to intensify during treatment phases, as they manage their own psychological distress while taking on additional caregiving responsibilities (Loeb et al. 2022; Collaço et al. 2018).

Existing literature consistently highlights the profound psychological impact experienced by partners who undertake caregiving roles during prostate cancer treatment (Jiang et al. 2024; Vartolomei et al. 2025). Caregivers frequently grapple with anxiety related to potential disease reoccurrence, depression and significant stress from managing complex caregiving responsibilities alongside personal obligations. Moreover, Levesque et al. (2015) reveal a persistent pattern of emotional isolation and unmet informational needs, often driven by partners' reluctance to openly express distress out of a desire to shield the patient emotionally. This self‐imposed silence, coupled with inadequate systemic support, contributes to caregiver burden, negatively impacting their mental and physical health, relationship dynamics and overall quality of life (Green et al. 2022; Collaco et al. 2021; Vyas et al. 2022). Addressing these unmet needs through targeted support and better understanding of partners' experiences could significantly improve patient and partner care quality (Green et al. 2022; Fox et al. 2019).

The physical side effects of prostate cancer treatments, such as incontinence and erectile dysfunction, have significant psychosocial implications for both patients and their partners. Some patients feel a sense of emasculation and loss of identity due to these effects, which in turn affect relational dynamics, intimacy and overall relationship satisfaction (Wang et al. 2022; Qian et al. 2022). On the other hand, partners who often struggle to discuss these issues openly may also feel neglected and isolated, compounding the distress brought on by their partner's cancer journey (National Health Service [NHS] 2021; Huang et al. 2023). These challenges highlight a critical need for comprehensive support systems that help partners navigate these sensitive topics, manage evolving relationship dynamics, and maintain open communication with their loved ones.

Psychological challenges are pervasive among partners, including anxiety related to disease recurrence, depression, and the stress of balancing caregiving with other life responsibilities. These stressors are amplified by societal stigma surrounding topics such as sexuality and ageing, making it challenging for partners to seek the support they need (Qian et al. 2022; Teo et al. 2019). People from sexual and gender minority groups are less likely to frequently attend screening, leading to a limited representation in prostate cancer research and a lack of culturally tailored support (Qian et al. 2022; Ma et al. 2021). Research into these experiences could help inform healthcare providers and policymakers about the importance of providing inclusive, equitable support to diverse groups of prostate cancer caregivers (Castro et al. 2024). Caregivers provide unpaid care to patients with prostate cancer, encompassing personal care, medication management, emotional support, appointment coordination, and advocacy, influenced by the recipient's condition, the caregiver's abilities, and available resources.

Partners play a substantial role in managing patients' day‐to‐day care and motivating them to engage in health‐promoting behaviours such as regular physical activity and emotional resilience strategies (Collaço et al. 2018; Green et al. 2022). Partners' involvement in patients' recovery is often instrumental to patients' wellbeing, highlighting the importance of partners' own physical and emotional health within the caregiving dynamic (Gandaglia et al. 2021). However, focusing largely on patient‐centred outcomes often leads to partners' contributions and needs being overlooked within healthcare settings. This oversight has led to a gap in support for partners, who may not receive adequate assistance from healthcare professionals or access to resources that address their mental health and wellness (Hedestig et al. 2005; Williams et al. 2014). Addressing this gap by integrating partner‐focused support could enhance the efficacy of prostate cancer care and improve outcomes for both parties.

Furthermore, the psychosocial demands on partners are accompanied by distinct challenges related to decision‐making around treatment options. Prostate cancer treatments, especially those involving surgery and hormone therapies, often necessitate critical choices, with partners playing an active role in these discussions. This decision‐making process can be fraught with uncertainties, as partners weigh potential treatment outcomes against side effects such as dysuria, nocturia, and reduced sexual function, all of which can affect long‐term quality of life (van Dijk et al. 2024; Chambers et al. 2018; Kerman et al. 2023).

Evidence suggests that shared decision making and transparent communication in these areas can foster better patient partner alignment and satisfaction with care, although many partners report feeling unprepared or unsupported in navigating these complex medical choices (van Dijk et al. 2024; Chambers et al. 2018).

The multifaceted impacts of prostate cancer highlight a compelling need for further research on the experiences of partners. This paper aims to address the gaps in understanding by examining the physical, emotional and relational challenges faced by partners of prostate cancer patients. A more comprehensive approach to support and interventions could significantly improve the overall quality of life for both patients and partners. This study aims to illuminate the unique needs of partners, whose health and wellbeing are vital to the effective support of prostate cancer patients. Understanding partners' experiences could transform support mechanisms in healthcare, moving toward holistic care models that recognise the interconnected nature of patient‐partner health dynamics.

## 2 Methodology

### 2.1 Research Design

To explore the lived experience of partners of men with prostate cancer in Ireland, a qualitative descriptive design (Doyle et al. 2020) was utilised to help enhance the depth and authenticity of the study. The utilisation of this design is also commonly employed by nursing and other healthcare research to offer a comprehensive insight into the specific and detailed phenomenon, particularly in areas with a knowledge deficit (Ayton 2023). A semi‐structured interview was conducted to help researchers build extensive follow‐up questions that will help obtain the needed results of the study.

### 2.2 Study Settings

The study was conducted in Ireland, involving participants recruited from outpatient oncology clinics and prostate cancer support networks like Men Shed, ICS, etc. Interviews were conducted in the preferred locations and means of participants (through phone call, zoom video call, or Microsoft Teams, private residents/community setting) to help maintain privacy and accessibility to the geographical area. Data collection took place between March 2024 and the end of February 2025.

### 2.3 Study Recruitment Process, Sampling and Sample Size

For this study, to capture a diverse range of experiences, an invitation letter and participant information leaflet via email were sent by the researchers to cancer groups and other relevant health organisations, such as the LGBTQ+ community, Men's and Women's Sheds, etc. These organisations provide crucial support networks to cancer patients, families and caregivers. It also offers Irish men and their partners a space to address various health and social concerns impacting both their lives and their communities. A poster and information leaflet were created and distributed among the public, and radio announcements were used to enhance outreach for recruitment.

### 2.4 Eligibility Criteria and Sample

The study included partners of men who were recently diagnosed, undergoing treatment, or in post‐treatment stages. However, partners who were critically ill or had cognitive impairments were excluded to ensure accuracy and reliability of the data. Due to this, the following Inclusion and Exclusion criteria were utilised in Table 1.

**TABLE 1:** Inclusion and exclusion criteria.

Inclusion criteria	Exclusion criteria
Partners of men living with prostate cancer at any stage	Partners who are not involved in any prostate cancer journey
Partners residing in Ireland	Partners outside Ireland
Partners who can communicate in English	Partner with cognitive impairment
	Partners who are clinically ill

### 2.5 Sampling and Sample Size

While the study was guided by purposive sampling principles to recruit partners with direct experience of prostate cancer caregiving, recruitment relied on self‐selection through community and advocacy organisations. As such, the final sample reflects a form of purposive convenience sampling. After interviewing nine (9) participants, no new data were gained from interviews, showing that sufficient information had been collected, and data saturation had been achieved. According to Guest et al. (2006), data saturation can occur with smaller samples in focused studies with similar participants. In this instance, data saturation was assessed during data collection by analysing each interview transcript for emerging themes. The fact that no new theme appeared after the ninth interview suggested that saturation was reached, bolstering the credibility of the findings despite the seemingly small sample size.

### 2.6 Data Collection and Tool

Before the interview began, the purpose of the study, participants' rights and consent, and the use of a voice recorder during the interview were communicated to participants, seeking approval and promoting awareness. A semi‐structured interview approach (see Appendix S1) was used to gather in‐depth information about the experiences and perspectives of partners of patients living with prostate cancer. Interviews were conducted by the lead investigators (S.M., O.D.) from the beginning of March 2024 to the end of February 2025, either face‐to‐face, virtually through Microsoft Teams, or by phone as per the participants' preference. Prior to the inception of the conversation, pretesting was done to assess and ensure the effectiveness of the interview questions. Participants were also asked about their hobbies and formal field of work to reduce stress, enhance free communication, and promote cooperation. To ascertain an in‐depth understanding of participant responses, follow‐up questions were introduced to probe for underlying reasons and motivations. Field notes and participant cues were also noted during the interview, which lasted between 30 and 40 min. For example, if the majority of participants highlighted an unexpected challenge like a decrease in intimacy, probing and subsequent interview questions were included to explore the challenge to help ascertain the challenge for them.

### 2.7 Data Analysis

Data analysis started immediately after the first interview was audio‐recorded and transcribed verbatim. The transcribed data were transferred to NVivo 12.0 software, which is designed to help in coding qualitative data. To acquire a thorough comprehension of each participant's narrative, the principal investigator (S.M.) carried out an independent iterative analysis of the transcript with an open exploratory approach. The study utilised a collaborative coding approach, with two researchers (O.D. and C.O.D.) independently coding a subset of interview transcripts. Researchers met to discuss and agree on a unified framework, ensuring an understanding of the data. Strategies like member checking and peer debriefing were implemented to enhance the trustworthiness of the findings.

**FIGURE 1:**
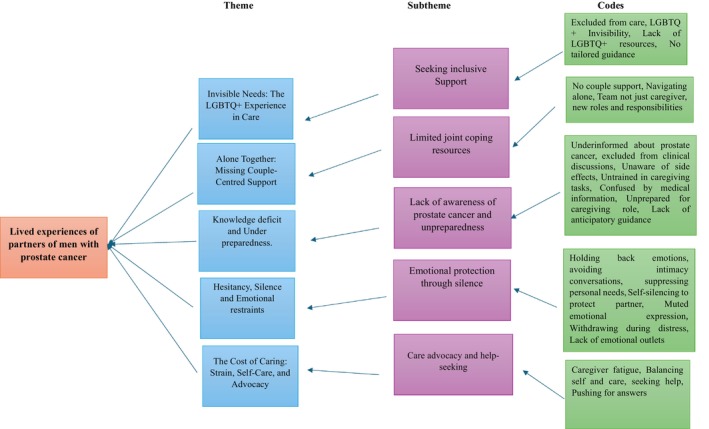
Thematic framework of partner experiences in prostate cancer care. This figure outlines the synthesis of qualitative data regarding the challenges faced by partners. Key themes include the physical and emotional ‘Cost of Caring’ involving fatigue and advocacy; ‘Knowledge Deficits’ regarding clinical and practical preparedness; and ‘Alone Together’, which highlights the lack of couple‐centred support and joint coping. Additionally, it captures ‘Hesitancy and Silence’ regarding intimacy and emotions and the ‘Invisible Needs’ of the LGBTQ+ community.

Following several transcript evaluations, a thematic analysis approach by Braun and Clarke (2024) was utilised to enhance topic development and promote a deeper understanding. Thematic analysis consists of six key phases: (1) Familiarisation of oneself to the data including taking notes and identifying area of interest, (2) Generating codes comes with systematically coding and assigning labelling to the segmented text, (3) Searching for themes involves critically examining and refining themes, (4) Reviewing themes ensures the themes are thoroughly investigated and support in alignment with data, (5) Defining and naming themes is when each theme is clearly defined, articulated, and given a concise and informative name that captures its essence and (6) Producing reports is the final phase which involves presenting the findings in a compelling narrative that addresses the research question. To guarantee credibility and coherence with the study's aim, the authors analysed the emergent significance from the analytical results, subthemes and themes (Figure 1). In practice, interviews were audio‐recorded, transcribed verbatim, and analysed using reflexive thematic analysis. Initial coding was conducted independently by two researchers, who met regularly to discuss and refine codes. Themes were developed iteratively through constant comparison across transcripts. NVivo 12.0 or manual coding was used to manage the data.

### 2.8 Ethics Statement

To ensure ethical standards are followed, the researchers applied for ethical approval from the University of Limerick ethics board. The study was reviewed and approved by the Faculty of Education and Health Sciences Research Ethics Committee at the researcher's university on March 13, 2024 (Reference No: 2023_12_10_EHS). The study adhered to strict confidentiality guidelines, using pseudonyms and avoiding direct quotes to protect participants' identities. The research design prioritised emotional safety, with participants fully informed of their voluntary participation and the right to withdraw at any time without consequence. The interview environment was comfortable and private, and interviewers were trained to be sensitive and empathetic. The study was also conducted in accordance with the Declaration of Helsinki. All participants provided written informed consent before participation.

Data were securely stored in accordance with the standard of University of Limerick data protection protocols, with hard copy and electronic files securely stored in locked cabinets. Access to audio recordings was restricted to principal researchers, whereas electronic transcripts were anonymised and retained securely for 7 years. Participants were debriefed at the end of the interview, thanked for their time and contribution, and informed about the study's dissemination. Support resources like the Irish Cancer Society counselling services were also provided to participants, providing them with information about relevant organisations to help address their grievances.

## 3 Results

### 3.1 Participants Characteristics

The study utilised diverse participants of partners of persons living with prostate cancer. Of the 15 targeted participants, nine (*n* = 9) participants were interviewed due to data saturation as indicated by Rahimi (2024). The study consisted of two black Irish participants and seven white Irish. The study also involved both male and female partners, thus male same‐sex couples (*n* = 4) and heterosexual couples (*n* = 5). The age range was between late 30s and early 70s, with a mean age of 55 years. The duration of their marriages was between 5 and 35 years, with two divorced (but still caring for their partners) and seven married. With regards to stages of their partners' diagnosis, two were early stage, five were intermediate stage, and two were advanced or recurrent stage. See Table 2 below for further details.

**TABLE 2:** Participants' demographics.

Participant	Type of relationship	Age range (years)	Gender	Ethnicity	Marital status and years	Stages of condition
P1	Same sex couples	35	Male	White Irish	Married (10 years)	Early stage
P2	Heterogeneous couples	55	Female	Black Irish	Married (20 years)	Intermediate stage
P3	Same sex couples	59	Male	White Irish	Married (24 years)	Intermediate stage
P4	Same sex couples	47	Male	White Irish	Married (9 years)	Intermediate stage
P5	Heterogeneous couples	70	Male	White Irish	Married (35 years)	Intermediate stage
P6	Heterogeneous couples	66	Female	Black Irish	Divorce	Advanced stage
P7	Heterogeneous couples	63	Female	White Irish	Divorce	Advanced stage
P8	Heterogeneous couples	49	Female	White Irish	Married (15 years)	Intermediate stage
P9	Same sex couples	42	Male	White Irish	Married (8 years)	Early stage

### 3.2 Major Themes

We identified five major themes with regard to the experiences of partners of men with prostate cancer. These themes are (1) Invisible Needs: Experience in Care, (2) Alone Together: Missing Couple‐Centred Support, (3) Knowledge deficit and under preparedness, (4) Hesitancy, Silence and emotional restraints and (5) The Cost of Caring: Strain, Self‐Care and Advocacy.

#### 3.2.1 Theme 1: Invisible Needs: Experience in Care

Female partners expressed concern about inadequate focus on emotional and mental health for caregivers, with emphasis often on physical conditions and limited acknowledgement of emotional struggles. Which often makes them feel overlooked, as one participant indicated that:

Most of the focus seemed to be on the patient, which is understandable, but as a caregiver, you are dealing with so much too. (p8)

LGBTQ + partners often experience a sense of invisibility and face unique challenges within the healthcare setting due to assumptions and lack of inclusive resources. A significant concern raised by male sex couples was the repeated need to clarify their relationship to healthcare professionals, leading to emotional burden. As one partner shared:

The doctors and nurses kept assuming I was his friend or brother, not his partner… it is really frustrating having to correct them at all times. (p3)

This constant need for explanation can be emotionally exhausting and undermine their recognised role as primary caregivers. Furthermore, participants reported feeling excluded from the support system designed solely for heterogeneous partners, highlighting the lack of tailored resources for the LGBTQ + community.

We want a support group, but it felt like it was for straight couples… No one talked about what it is for people like us. (p9)

This sense of isolation is compounded by the scarcity of resources addressing the specific impact of prostate cancer on same sex relationships, forcing partners to navigate these challenges without adequate guidance. This, according to Melies' Transition Theory, is an example that makes it difficult for partners to make a healthy transition.

#### 3.2.2 Theme 2: Alone Together: Missing Couple‐Centred Support

Despite being intimately involved in their partner's care, many partners feel excluded from medical discussion and lack support that addresses couples' shared experiences, resulting in a hindrance to navigating patient care and post‐treatment adjustment as a united front. One partner emotionally asked:

There was a lot of support for him… but there were none of any support for both of us together? (p1)

This lack of couple centred support hindered their ability to navigate the complexities of patient care and post‐treatment adjustment as a united front. The absence of guidance on managing the emotional impact as a couple left partners feeling isolated in their shared experiences. Subsequently, one participant noted that:

We had to navigate all the challenges alone… there was no guidance on how to deal with the emotional toll as a couple. (p3)

The lack of inclusion in educational discussion and treatment planning left partners feeling ill‐equipped and compelled to seek information independently. This kind of experience highlights a critical deficit in the conditions necessary for a smooth and healthy transition for couples.

#### 3.2.3 Theme 3: Knowledge Deficit and Under Preparedness

This theme highlighted that almost all the partners had no or limited prior understanding of the condition of prostate cancer, its symptoms, and management. This lack of initial knowledge often led to a sense of being overwhelmed following their partner's diagnosis. For instance, one partner admitted that:

I did not really know what prostate cancer meant. I just assumed it was treatable, and we would move on, but it's changed everything. (p2)

In some cases, a lack of awareness about early warning signs of prostate cancer resulted in delayed recognition and seeking medical attention. One participant recounted that:

I thought he had a lot of urine frequency… but I thought it was maybe a natural process that he would go through. (p7)

Additionally, partners expressed confusion regarding treatment options and their potential long‐term consequences, often finding the information provided by professionals to be too technical or primarily focused on the patient perspective, leaving them feeling underprepared for the realities of post‐treatment life.

Nobody really told me what incontinence would actually be like… I was not ready for pads, the accidents or the embarrassment. (p4)

This widespread knowledge/information and readiness deficit unswervingly aligns with the Melies concept of inadequate preparedness for the development and situational transition of cancer care.

#### 3.2.4 Theme 4: Hesitancy, Silence and Emotional Restraints

This theme explores the reluctance of partners to openly discuss sensitive areas like sexual changes and emotional distress, often stemming from a desire to protect their partner's dignity and masculinity. Hesitancy was sometimes evident in their communication style during interviews. The impact of these unspoken issues on the relationship was significant, as illustrated by one partner who stated:

We never really talked about how our sex life has changed… I did not want him to feel worse. (p3)

The difficulty in initiating these conversations often left emotional and physical changes unaddressed, creating a sense of distance. As another participant shared:

Even now I do not know the right words. Somethings are just left unsaid. (p7)

This culture of silence and self‐censorship among partners highlights a need for a more open communication pathway and support in addressing these sensitive aspects of living with prostate cancer. Similarly, such behaviours mirror a pattern of response in the Melies theory that can be problematic, hindering emotional integration and adaptation during the caregiving transition.

#### 3.2.5 Theme 5: The Cost of Caring: Strain, Self‐Care and Advocacy

The demand of caregiving often led to significant personal strains, highlighting the critical need for self‐care and proactive advocacy for support. Partners frequently describe neglecting their own wellbeing, leading to burnout and emotional exhaustion, often early in the caregiving journey. One partner expressed his overwhelming feeling stating:

I believed I had to manage all caregiving responsibility independently, which quickly became overwhelming. (p5)

This emotional toll could be so profound to the extent of making one participant recount:

I did not realise how drained I was until I could not stop crying one day. (p3)

Despite these challenges, the theme also encompasses the active seeking of support and information, recognising the importance of self‐preservation. As another partner reflected:

Asking for help was hard, but once I did, everything felt a bit more manageable. (p1)

This highlights the dual experiences of caregiver's burden and the eventual recognition of the necessity for support and self‐advocacy. To aid a healthy transition, it is crucial to actively seek coping mechanisms and negotiate these stressors, as this is related to the pattern of response and the need for nursing therapeutics.

## 4 Discussion

This study employed a qualitative descriptive study to explore the experiences and perspective of partners of men with prostate cancer in Ireland. As part of a comprehensive analysis of health illness transitions experienced by partners, Melies' Transition Theory (2010) will also be utilised. The experiences shared by partners brought about the development of five themes. To further understand how to intervene and facilitate healthy transitions, the themes were examined in relation to policy and health system context. Participants were primarily couples willing to engage in joint discussion, which may indicate relatively higher levels of relationship functioning. Couples experiencing significant emotional distance or relationship strain may have been underrepresented. As such, the findings should not be generalised to all couples affected by prostate cancer, and couples centred interventions may not be appropriate in all cases.

Partners of male same sex couples with prostate cancer frequently reported difficulty in navigating healthcare systems structured around heteronormative assumptions, which often rendered their relationship invisible or misunderstood (Panken et al. 2024). The design and delivery of quality of care frequently presume that all patients are heterosexual and partnered with women, creating barriers to inclusive engagement (Vartolomei et al. 2025). These conditions, grounded in cultural norms and systemic practices, reflect what Meleis' theory defines as conditions of transition factors that influence how individuals experience change and adjustment. Participants described emotional discomfort during clinical interactions, particularly when required to disclose their relationship status to providers who failed to demonstrate LGBTQ+ cultural competence. Such interaction contributed to patterns of responses characterised by pattern exclusions, relational withdrawal and reduced access to information critical for caregiving (Rosser et al. 2022). Panken et al. (2024) further note that many same sex partners avoid joint clinical visits due to prior experiences of discrimination, which diminishes opportunities for shared decision‐making and psychological support. These patterns often lead to increased psychological distress and decreased quality of life (Xu et al. 2023). Healthcare providers are pivotal in addressing these disparities. Inclusive and gender‐neutral communication practices, such as directly inquiring about partner involvement and avoiding gendered assumptions, are key nursing therapeutics that can facilitate more equitable care (Pratt‐Chapman et al. 2020). These strategies not only enhance transition outcomes but also improve relational wellbeing. At a system level, policy interventions are required to institutionalise inclusive practise. Recommended measures include LGBTQ+ inclusive consultation protocol and reforms to patient intake documentation to recognise diverse relationship structures (Ussher et al. 2023; European Commission 2020).

A significant finding that emerged from the study was the persistent absence of structured support for couples navigating prostate cancer together. This gap highlights a critical lack of couple care in prostate cancer management (Johnson et al. 2021; Mumuni et al. 2024). Although prostate cancer is regarded as a couple centred illness given its substantial impact on both the patient and partner, a significant number of participants reported that healthcare institutions exclusively focus on the patient, leaving partners to confront emotional and logistical challenges alone. Post‐treatment complications such as urinary incontinence, hormonal imbalances, and erectile dysfunction can significantly strain intimacy, trigger emotions and foster isolation within relationships (Charlick et al. 2024). These outcomes underscore the role of the healthcare environment as a barrier to effective transition, particularly when collaborative, inclusive care approaches are absent (Pratt‐Chapman et al. 2020). This reflects what Meleis' theory identifies as adverse conditions for transition, where lack of structural and relational inclusivity disrupts adjustment. The lack in many Irish healthcare institutions of couple‐oriented frameworks in the cancer care setting exacerbates these challenges (McConkey and Holborn 2018). For example, while the Irish Cancer Care Programme (NCCP) acknowledges the psychological burden of prostate cancer, current survivorship and clinical pathways rarely formalise partner inclusion in routine care (Health Service Executive [HSE] and National Cancer Control Programme [NCCP] 2025). Despite these challenges, some couples demonstrated positive adaptive strategies. Several participants describe developing deeper emotional intimacy through open dialogue, mutual reassurance and pursuit of shared activities that fostered connection. These responses are consistent with findings by Daniels et al. (2023) and Dave et al. (2024), suggesting that timely, inclusive communication from clinicians improves emotional resilience and understanding among cancer treatment couples, highlighting the potential for healthy relational transformation. However, Vartolomei et al. (2025) indicated that many partners developed private coping strategies without guided therapeutic support, revealing a systemic failure to engage relational units, especially in a survivorship context, without guided therapeutic support. To address these challenges, the Irish healthcare system needs to restructure service delivery to recognise patients and their partners as a unit of care (with patient consent), integrating partner‐inclusive practices into clinical care pathways. For example, the use of the Rapid Access Prostate Clinic (RAPC) creates a meaningful sustenance for relational education, consultation and posttreatment support (Health Service Executive [HSE] 2023). Nurse‐led survivorship programmes could be improved by incorporating partners in dialogue, intimacy challenges, enhancing informational equity and developing tailored educational resources to address couples based on concerns (Health Service Executive [HSE] and National Cancer Control Programme [NCCP] 2025). The HSE psycho oncology initiative should include couple‐based care modules and promote inclusive communication, which will encourage clinicians to use non‐gendered language and understand diverse relationship dynamics (Pratt‐Chapman et al. 2020; Patel et al. 2024). This potential underrepresentation may also influence perceptions of couple‐centred support, as couples experiencing high conflict, emotional distance, or relational strain may have different support needs that are not captured in the present findings. Policy reforms should include couples' needs assessment and outcome measurement tool in prostate cancer care, aligning service evaluation with Meleis nursing therapeutic framework (Ussher et al. 2023; European Commission 2020).

Additional findings revealed that 8 out of 9 partners of men with prostate cancer began their caregiver role with limited guidance or preparation, underscoring a profound knowledge gap and a significant lack of preparedness. This absence of foundational knowledge regarding the stages of prostate cancer, its symptoms, and potential complications intensified partners' emotional burden and contributed to feelings of uncertainty and inadequacy in their ability to provide care (Vartolomei et al. 2025; Charlick et al. 2024). These challenges correspond with what Meleis' theory identifies as an adverse condition of transition, where insufficient informational and structural resources hinder effective adaptation to a new caregiving role (Meleis 2010). Corresponding evidence from Owoo et al. (2022) and Ninnoni and Owoo (2023) similarly illustrates that poor preparedness not only elevates caregiver anxiety but also impairs confidence in clinical decision‐making, particularly when managing side effects or emotional reactions post‐treatment. In some cases, participants recounted making harmful or misinformed decisions in their caregiving efforts, further reinforcing the view of prostate cancer as a relational condition where the needs of the partners must be considered alongside those of the patient. This highlights the urgent need for inclusion of structured partner education within prostate cancer treatment policy and practice framework (Health Service Executive [HSE] and National Cancer Control Programme [NCCP] 2025). From a nursing therapeutics perspective, the integration of digital and interdisciplinary resources is essential to enhance caregivers' confidence during the transitional phase. Tools such as telehealth consultations, partner‐specific informational leaflets, and facilitated access to interdisciplinary care teams can increase engagement, reduce anxiety, and promote informed participation through the care continuum (Panken et al. 2024; Pratt‐Chapman et al. 2020). Regarding education, cancer support centres should extend their programming beyond the patient to include dedicated partner support. Literature by the European Commission (2020) and Patel et al. (2024) also indicates that interventions like awareness campaigns (on early signs and symptoms), pre‐treatment workshops, hospital discharge training, and eHealth literacy support help non‐clinical caregivers or partners navigate the caregiving transition with competence and emotional resilience, thus aligning with Meleis transition patterns, properties and conditions.

Seven out of nine participants in the study expressed reluctance to disclose their emotional concerns, resulting in a pattern of self‐imposed silence aimed at protecting their partners. This tendency toward emotional suppression reflects a coping strategy rooted in relational loyalty but often results in unaddressed psychological distress. Collaço et al. (2018) found that partners of men with prostate cancer frequently conceal their own fears, anxiety, and vulnerability in an effort to preserve relationship stability and safeguard the emotional wellbeing of the patient. Although this may initially serve as a form of emotional buffering, the long‐term effects can be destructive to both caregiving quality and relational intimacy (Green et al. 2022). This emotional restraint may be further amplified by the stigmatisation of same sex relationships in healthcare settings and by traditional norms of masculine stoicism, both of which inhibit open emotional expression (Vartolomei et al. 2025; Panken et al. 2024).

Studies indicate that partners who refrain from seeking help or voicing their struggles are more susceptible to caregiver burnout, depression and diminished wellbeing (Charlick et al. 2024; Patel et al. 2024). A longitudinal study conducted in Sweden demonstrated that partners of men with high‐risk prostate cancer experienced persistent anxiety and depressive symptoms lasting over a decade post diagnosis (Crump et al. 2024), illustrating the enduring mental health consequences of emotional suppression. As emotional restraint becomes chronic, it contributes to maladaptive patterns of response, as conceptualised in Meleis Transition Theory. In this context, emotional avoidance obstructs the cognitive and emotional integration required for a healthy caregiving transition (Meleis 2010). Prolonged silence and avoidance not only erode the psychological health of the partner but also strain communication and intimacy within the couple. Participants describe increased emotional distance, misunderstanding, and a shift toward platonic relationship dynamics, further compounding caregiving burden. Facilitating emotional communication is therefore essential to fostering adaptive transition. Healthcare providers must cultivate clinical environments that normalise discussions around intimacy, fear and psychological vulnerability. This includes explicitly encouraging partners to express their concerns, validating the emotional complexity of caregiving experience (European Commission 2020). Partner inclusive communication training focusing on oncology teams to reduce avoidance of behaviours and promote emotional resilience, whereas structured support services like couples counselling and peer support groups should be regularly embedded within survivorship care modules (Health Service Executive [HSE] and National Cancer Control Programme [NCCP] 2025). At the policy level, guidelines should include partner inclusion in psychological assessment, care planning, counselling, sexual health support and mental health screening for prostate cancer patients, improving psychological outcomes and relational wellbeing to enhance the quality of life for couples managing the illness trajectory (Vartolomei et al. 2025; Ussher et al. 2023).

The study found that many partners tend to neglect their own physical and emotional health while caregiving, resulting in diminishing wellbeing, chronic fatigue, heightened stress, and a persistent sense of being overwhelmed. This pattern of neglect reflects an internalised belief that their own needs are secondary to those of the patient. Supporting evidence from recent studies confirms that caregivers of men with prostate cancer often report extreme exhaustion, exacerbated by complex patient care demands and the psychological toll of long‐term vigilance (Panken et al. 2024). This cumulative effect of prolonged stress, emotional suppression, and disruptive routines negatively impacts both the caregiver and the patient, especially when seeking support is perceived as emotionally burdensome or socially stigmatised (Charlick et al. 2024; Patel et al. 2024). Meleis Transition Theory helps to frame these experiences, highlighting how failure to prioritise caregiver self‐care represents a breakdown in patterns of response that are critical for healthy adaptations (Meleis 2010). Personal and environmental factors like limited access to support services, financial constraints, and the stigma surrounding help‐seeking can exacerbate caregiver burden and restrict wellbeing during transition (Charlick et al. 2024). Some partners, however, reframed self‐care as a functional necessity rather than a discriminatory act, recognising that their own emotional stability was essential for sustained caregiving. Partners who engage in restorative activities or who accessed a supportive network often described greater vitality, improved mood and enhanced relational patience. Transitioning from self‐neglect to self‐care frequently requires access to appropriate knowledge, tools and support structures. When carers are equipped with stress management techniques, communication strategies, or access to community‐based services, they are better positioned to navigate the demands of caregiving without compromising their own health (Li et al. 2020). Family‐led interventions, such as respite conditions and psychological support, were also deemed valuable in helping partners reorient toward their own wellbeing (Roberts et al. 2024). Clinically, healthcare professionals should monitor caregivers' wellbeing during follow up appointment and promote self‐care strategies like stress reduction techniques and referral to support groups or respect services (Vartolomei et al. 2025). The use of groups like Women's Shed, LGBTQ+ support groups, the Women Collective Ireland (WCI) and Irish Cancer Society to help resolve any mental, psychological or emotional challenges should be encouraged. Health systems and policy frameworks should institutionalise caregiver support through educational programs; partners should support and formalise screening protocols to enhance self‐efficacy and resilience. At the structural level, policies that provide financial caregivers leave, and employment protection can eliminate practical barriers to self‐care, making it possible for partners to sustain their caregiving role without risking income, stability or health (European Commission 2020; Health Service Executive [HSE] and National Cancer Control Programme [NCCP] 2025).

Clinically, healthcare providers should receive compulsory training in inclusive, couple centred communication and supportive counselling techniques. In accordance with the HSE's National Cancer Strategy 2017–2026 and its Model of Care for Psycho‐Oncology 2020 specialised training modules, incorporating and addressing LGBTQ+ relationship dynamics, should be mandatory for health professionals managing prostate cancer patients and their partners (Charlick et al. 2024; Health Service Executive [HSE] 2023). The European Commission's LGBTQ+ Equality strategy 2020–2025 also calls for a healthy system across member states to provide culturally competent care, underscoring that the policy is imperative to ensure non‐discriminatory, relationship‐inclusive practice at the point of delivery (European Commission 2025). Institutional policies should therefore mandate routine partner involvement in prostate cancer care pathways, require the systematic assessment of caregivers' distress through an evidence‐based screening tool (as endorsed by HSE psycho‐framework), and foster an LGBQT+ affirming environment through structured intervention, inclusive resources, and protocol‐driven staff training. These policy recommendations align with the European Union's Council Conclusions on Access to Inclusive and Quality Health Care 2021, which emphasise equality (Ussher et al. 2023; European Commission 2025).

Educational institutions, particularly those training future health professionals, should integrate comprehensive teaching modules on inclusive care practices into undergraduate and postgraduate curricula. The Nursing and Midwifery Board of Ireland (NMBI) requires that accredited programmes prepare practitioners; thus, explicit instruction on LGBTQ+ family systems, relational dynamics, and couple centred methodologies should be formalised within academic standards (Nursing and Midwifery Board of Ireland [NMBI] 2023; European Commission 2025). This study's small, geographically specific sample limits generalisability and transferability of findings. Moreover, data obtained through self‐reports could introduce biases related to recall accuracy or social desirability, potentially impacting the findings' precision. Future studies would benefit from larger, diverse samples and longitudinal designs to broaden applicability. As researchers, we recognise potential biases due to our academic and professional backgrounds in health sciences, particularly nursing. To minimise these biases, we maintained reflexive journals, engaged in peer debriefing, and critically reflected on data analysis processes to uphold analytical integrity.

This study provides a crucial insight into the multifaceted challenges faced by partners of men with prostate cancer, particularly highlighting the often‐invisible struggles of LGBTQ+ partners and the pervasive lack of couple centred support. Our findings extend existing knowledge by explicitly linking these experiences to barriers to healthy transitions within the healthcare system, emphasising the need to move beyond an individual‐focused model of care. The study raises critical questions about how healthcare systems can become more inclusive, proactively support couples navigating illness together, and adequately prepare partners for their caregiving roles. These findings align with previous work highlighting the heterogeneity of couples' support needs in prostate cancer care (Chambers et al. 2019; Ugalde et al. 2019). Recent evidence on couple‐based and caregiver‐focused interventions was integrated to contextualise the findings and inform recommendations. Although participants in this study expressed a clear absence of couple‐centred support, it is important to recognise that such approaches may not be appropriate or effective for all couples. Evidence from intervention studies suggests that the benefits of couple‐based care are contingent on relationship quality, emotional intimacy, and readiness to engage jointly (Chambers et al. 2019; Li et al. 2020). Couples experiencing long‐standing relational strain, unresolved conflict, or limited emotional closeness may find joint interventions challenging or burdensome, particularly when sensitive issues such as sexuality, role changes, or emotional distress are addressed. In these contexts, caregiver‐focused or individualised psychosocial interventions may be more appropriate and supportive (Ugalde et al. 2019; Dave et al. 2024). These findings highlight the need for flexible, tailored support models that avoid one‐size‐fits‐all approaches and instead offer stepped or choice‐based pathways aligned with couples' relational contexts and support needs. Future research should focus on developing and evaluating interventions that address these identified gaps, ultimately aiming to improve the quality of life for both patients and their partners.

### 4.1 Limitation

This study has several limitations that may affect the credibility and transferability of its findings. First, the small sample size and geographic focus on partners in Ireland limit the extent to which results can be generalised to other cultural or healthcare contexts. Additionally, the voluntary nature of participation may have introduced self‐selection bias, potentially underrepresenting individuals who are less emotionally expressive or more reluctant to engage in reflective conversations. Variability in interview modalities conducted via face‐to‐face, telephone, and virtual platforms may have influenced data depth and consistency. For instance, non‐verbal cues and rapport‐building opportunities are generally richer in face‐to‐face interviews, potentially affecting the quality of responses. Furthermore, the use of convenience sampling may have introduced selection bias, as participants who volunteered were likely those more comfortable discussing relationship and intimacy issues. Consequently, couples experiencing significant emotional distance or relationship distress may be underrepresented, and the findings may not reflect the full range of couples' experiences following prostate cancer. Given the voluntary nature of participation, couples experiencing significant emotional distance, conflict, or relationship distress may have been less likely to participate, and their support needs may differ substantially. As such, the applicability of couple‐centred care approaches identified in this study may be limited for some groups, reinforcing the need for alternative or individualised caregiver‐focused interventions. Social desirability bias may also have influenced participants' narratives, especially when discussing sensitive topics such as relationship dynamics, sexuality, or caregiving burden. To mitigate bias, the research team engaged in reflexivity throughout the study. However, the positionality of the researchers, two males and one female, all with backgrounds in nursing, may have shaped both the interpretation and framing of data. Although professional expertise can enhance clinical insight, it may also carry implicit assumptions about caregiving roles and relational dynamics. Finally, this study's qualitative design prioritises depth over breadth. Future research could benefit from the inclusion of larger, more diverse samples across cultural contexts and from employing mixed methods to triangulate findings and examine the broader applicability of couple‐centred and inclusive care practices in prostate cancer management.

## 5 Conclusion

This descriptive study shed light on the overlooked experiences of partners of men with prostate cancer in Ireland. Particularly for LGBTQ+ people who often feel invisible and marginalised, the current healthcare system sometimes falls short in helping partners. As couples are driven to clarify their relationships and independently search for information, this results in great emotional weight and tiredness. Lack of couple centred support makes partners unprepared to negotiate the difficult emotional and pragmatic terrain of patient care, excluding them from important medical decisions. The discovered knowledge gaps and resulting unpreparedness for caregiving, together with great hesitancy and emotional restraints in addressing sensitive issues, combine to produce a high cost of caring that presents as strain and burnout. Ignoring the needs of partners not only affects their personal well‐being but also influences communication, intimacy, and general quality of life for the couple, thereby impeding the patient's recovery and the couple's capacity to reach a good transition across the cancer path. Ignoring these needs runs the danger of aggravating carer stress, lowering treatment plan adherence, and endangering the general welfare of the family. Nursing therapeutics should give top priority to establishing suitable environments for emotional expression, providing couple‐focused educational sessions, and integrating digital resources including telehealth and partner‐specific information. Healthcare professionals must receive dedicated training in inclusive, gender neutral, and couple centred communication skills to address these urgent gaps. Beyond scattered support, health systems and policy makers have to advocate funding for caregiver‐specific services, create guidelines for regular partner inclusion in care planning, and create LGBTQ+ friendly structures and resources within cancer support services. Understanding that survivorship depends on partners will help to change prostate cancer treatment, enhancing patient wellbeing and outcomes as well as those of supporters.

## Author Contributions

The study was designed by S.M., with data collection and analysis by S.M., C.O. and O.D., and the final manuscript was drafted, revised, and approved by all authors. All authors have read and agreed to the published version of the manuscript.

## Funding

The authors have nothing to report.

## Ethics Statement

Ethics approval for this study was granted by the Faculty of Education and Health Sciences Research Ethics Committee at the researcher's university on 13 March 2024 (Reference No: 2023_12_10_EHS).

## Consent

Consent was obtained from all participants involved in the study.

## Conflicts of Interest

The authors declare no conflicts of interest.

## Supporting information


**Appendix S1:** nop270585‐sup‐0001‐AppendixS1.docx.

## Data Availability

The qualitative data generated and analysed during the current study are not publicly available due to ethical approval requirements and the need to protect participant confidentiality. In accordance with the conditions of ethics committee approval and informed consent procedures, individual‐level data cannot be shared openly. However, de‐identified data may be made available from the corresponding author upon reasonable request.
