# Supplementary material for: The Experiences and Perspective of Partners of Men With Prostate Cancer in Ireland: A Qualitative Descriptive Study
Source: Nurs Open. 2026 May 5;13(5):e70585. doi: 10.1002/nop2.70585 (PMC13139899; doi:10.1002/nop2.70585)
Supplement: Supplementary file 1 — Appendix S1: nop270585‐sup‐0001‐AppendixS1.docx. [file NOP2-13-e70585-s001.docx]

**Appendix**

**Appendix 1: Interview guide (Partners of persons with prostate cancer)**


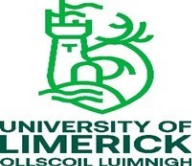


**Introduction**

Good morning/afternoon my name is Seidu Mumuni. Thank you for agreeing to take part in this interview. The purpose of this study is to explore the experiences of partners of men living with prostate cancer, including emotional, relational, and caregiving experiences. There are no right or wrong answers. We are interested in your personal experiences and perspectives. Everything you share will be kept confidential, and you may choose not to answer any question or stop the interview at any time. With your permission, the interview will be audio-recorded to ensure accuracy. On occasions I may take notes so that I do not miss anything you have to say. The interview will last approximately 40-60 minutes.

**Interview Guide**

**Background and Context**

1. Can you tell me a little about yourself and your relationship with your partner?
2. At what stage of prostate cancer is your partner currently (e.g., diagnosis, treatment, post-treatment, recurrence)?
3. Can you describe your role in supporting or caring for your partner since the diagnosis?

**Experience of Diagnosis and Early Transition**

1. Can you tell me about when your partner was first diagnosed with prostate cancer?
2. How did you personally feel during the period following the diagnosis?
3. Did you feel prepared for what lay ahead at that time?

**Caregiving Role and Preparedness**

1. How has your role as a partner or caregiver changed since your partner’s diagnosis?
2. What kinds of responsibilities have you taken on?
3. Did you feel equipped or supported to take on these responsibilities? Why or why not?
4. Were you given information or guidance specifically for partners or caregivers?

**Experiences with Healthcare Services**

1. How would you describe your experiences with healthcare professionals during your partner’s care?
2. To what extent did you feel included in consultations or decision-making?
3. Did you ever feel overlooked or invisible within the healthcare setting?
4. For participants in same-sex relationships or from minority backgrounds:
5. Did you feel your relationship was recognised and respected within healthcare settings?

**Couple-Centred Support and Relationship Impact**

1. How has prostate cancer affected your relationship as a couple?
2. Prompt: Emotional closeness
3. Prompt: Communication
4. Prompt: Roles and dynamics
5. Were there any supports offered that focused on you as a couple rather than only on the patient?
6. How did you and your partner manage challenges together?
7. Prompt: What helped?
8. Prompt: What made things harder?
9. Do you feel that couple-centred support would have been helpful for you? Why or why not?

**Section 6: Intimacy, Emotional Expression, and Silence**

1. How has your partner’s diagnosis or treatment affected intimacy or closeness in your relationship?
2. How comfortable did you feel discussing sensitive topics (e.g., sexuality, emotional distress)?
3. Prompt: With your partner
4. Prompt: With healthcare professionals
5. Were there things you chose not to talk about?
6. Prompt: What influenced that decision?
7. How did holding back or expressing emotions affect you personally?

**Section 7: Impact on Your Wellbeing**

1. How has supporting your partner affected your own emotional or physical wellbeing?
2. Did you feel able to prioritise self-care during this time?
3. Prompt: What got in the way?
4. Prompt: What helped?
5. Did you ever seek support for yourself?
6. Prompt: From whom?
7. Prompt: What was that experience like?

**Section 8: Coping, Adaptation, and Transition**

1. Looking back, what helped you adapt to your caregiving role?
2. Were there moments when you felt overwhelmed or unsupported?
3. Prompt: How did you manage those times?
4. Have your needs changed over the course of your partner’s illness?

**Section 9: Reflections and Recommendations**

1. What do you think healthcare professionals should better understand about partners of men with prostate cancer?
2. What kinds of support would have made the biggest difference for you?
3. Do you think all couples benefit from couple-centred care, or should support be tailored differently?
4. Prompt: Why?
5. Is there anything else about your experience that you feel is important and has not been discussed?

**Closing Script**

Thank you very much for sharing your experiences. Your contribution is extremely valuable and will help inform more inclusive and supportive care for partners of men living with prostate cancer. If discussing these topics has raised any concerns or emotions for you, support resources are available, and we can provide information if you wish.
